# Tracking Nile Delta Vulnerability to Holocene Change

**DOI:** 10.1371/journal.pone.0069195

**Published:** 2013-07-29

**Authors:** Nick Marriner, Clément Flaux, Christophe Morhange, Jean-Daniel Stanley

**Affiliations:** 1 Centre National de la Recherche Scientifique, Laboratoire Chrono-Environnement, Université de Franche-Comté, Besançon, France; 2 Centre National de la Recherche Scientifique, Centre de Recherche et d’Enseignement de Géosciences de l’Environnement, Aix-en-Provence, France; 3 Université Aix-Marseille, Centre de Recherche et d’Enseignement de Géosciences de l’Environnement, Aix-en-Provence, France; 4 Geoarchaeology Program, National Museum of Natural History, Smithsonian Institution, Washington, D.C., United States of America; Utrecht University, Netherlands

## Abstract

Understanding deltaic resilience in the face of Holocene climate change and human impacts is an important challenge for the earth sciences in characterizing the full range of present and future wetland responses to global warming. Here, we report an 8000-year mass balance record from the Nile Delta to reconstruct when and how this sedimentary basin has responded to past hydrological shifts. In a global Holocene context, the long-term decrease in Nile Delta accretion rates is consistent with insolation-driven changes in the ‘monsoon pacemaker’, attested throughout the mid-latitude tropics. Following the early to mid-Holocene growth of the Nile’s deltaic plain, sediment losses and pronounced erosion are first recorded after ~4000 years ago, the corollaries of falling sediment supply and an intensification of anthropogenic impacts from the Pharaonic period onwards. Against the backcloth of the Saharan ‘depeopling’, reduced river flow underpinned by a weakening of monsoonal precipitation appears to have been particularly conducive to the expansion of human activities on the delta by exposing productive floodplain lands for occupation and irrigation agriculture. The reconstruction suggests that the Nile Delta has a particularly long history of vulnerability to extreme events (e.g. floods and storms) and sea-level rise, although the present sediment-starved system does not have a direct Holocene analogue. This study highlights the importance of the world’s deltas as sensitive archives to investigate Holocene geosystem responses to climate change, risks and hazards, and societal interaction.

## Introduction

A key challenge concerning continental rivers is to better understand past, present and future river fluxes in the face of climate shifts, land-use alterations, river catchment modifications and their impact upon base-level geosystems [1-3]. Within this context, delta fronts are particularly sensitive recorders of global change because their sedimentary basins have sequestered rich environmental information at the terminus of the source-to-sink sediment conveyor [4-6]. Furthermore, deltas have been preferred areas of human occupation throughout the Holocene, nurturing the agricultural innovation, social organization and cultural exchange that led to the emergence of early complex societies [7-13]. Today, it is estimated that deltas host nearly half a billion people [14], engendering a series of environmental pressures that have sharpened focus on the resilience of these sensitive geosystems to future change [15-17].

The recent worldwide degradation of deltaic wetlands is often highlighted as an expression of global warming and human impacts [14]. For the instrumental period, sediment mass balance studies have greatly improved understanding of the link between natural and anthropogenic forcing factors in collectively mediating the fate of the world’s deltas [18]. Whilst human activities have increased fluvial sediment supply, the net amount of sediment reaching the ocean has actually decreased by ~10% through infrastructure projects such as dams and reservoirs [6,19]. These changes in sediment flux have led to significant coastal retreat, particularly in deltaic areas, and underscore the importance of understanding source-to-sink sediment conveyors at a variety of spatial and temporal scales [20]. Many studies of present delta systems have addressed the ability of deltaic wetlands to keep pace with sea-level rise, based on accretion status at decadal or shorter timescales and their comparison with sea-level rise as measured by tide gauges [21]. In a key study of 33 of the world’s most important deltas, Syvitski *et al*.[14]. found that 85% of deltaic areas have experienced severe flooding over recent decades, with forecasts suggesting that the area of vulnerable land will increase by about 50% in the next 40 years. The societal problems associated with this scenario are compounded by exponential demographics, particularly in the developing world [22]. By contrast, deltaic resilience in the face of longer-term Holocene changes has only been partly explored, despite its potential importance in characterizing the full range of present and future wetland responses. The Nile Delta represents a unique opportunity to fill this knowledge gap because robust chronostratigraphic, subsidence, sea level, palaeoclimate and sediment supply frameworks are now available to explore when and how its accretionary status has evolved during the Holocene [23-28].

**Figure 1:**
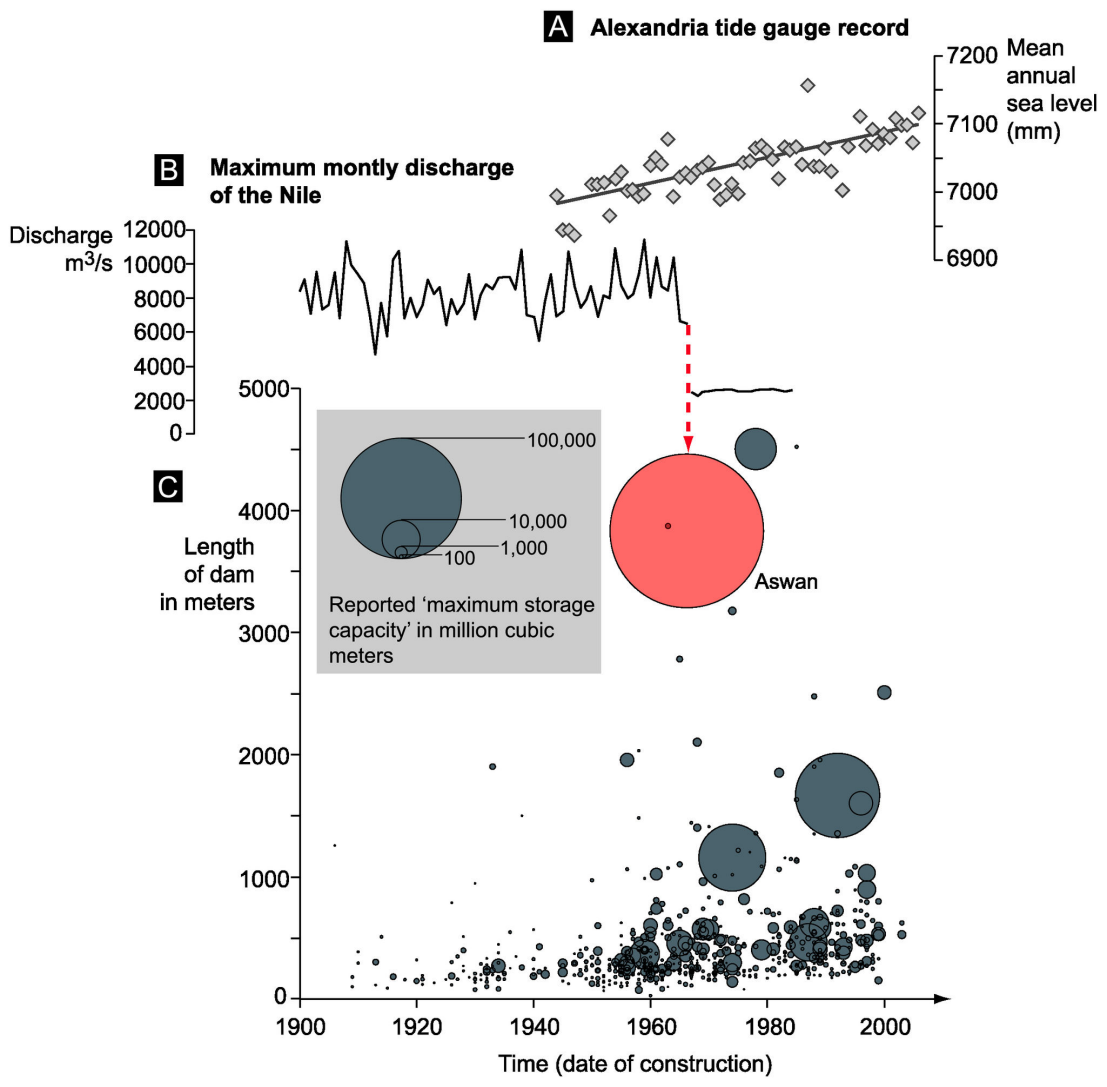
(A) Alexandria tide gauge record for the period 1944 to 2006 (data from reference [65]). (B) Maximum monthly discharge of the Nile during the 20^th^ century [66]. (C) Bubble plot of dams from the circum Mediterranean and southern Europe (data from reference [67]). The Aswan High Dam is represented in red. This figure shows the impact of the construction of the Aswan dam on Nile discharge and, indirectly, a drastic decrease in sediment input to the delta area. In the current context of rising Mediterranean sea level, locally attested by the tide gauge at Alexandria, this fall in alluvium does not allow the delta system to naturally offset sea-level change.

The Nile is the world’s longest river (>6500 km) and shaped the development of numerous complex societies, providing a reliable source of water for farming and linking populations between sub-Saharan Africa and the Mediterranean [29,30]. Its deltaic system lay at the heart of ancient Egyptian civilization and therefore understanding modifications in the delta’s geomorphology and accretionary status is particularly pertinent in interpreting its rich archaeological record. In recent decades, the Nile Delta has attracted considerable research interest as fears of reduced discharge, dwindling sediment supply, subsidence and projected sea-level rise potentially threaten one of Egypt’s most valuable economic resources and the future livelihood of more than 50 million people ([31,32]; Figure 1). This fragility has prompted the Intergovernmental Panel on Climate Change (IPCC) to assign the delta to its ‘extreme’ category of vulnerability hotspots [33].

**Figure 2:**
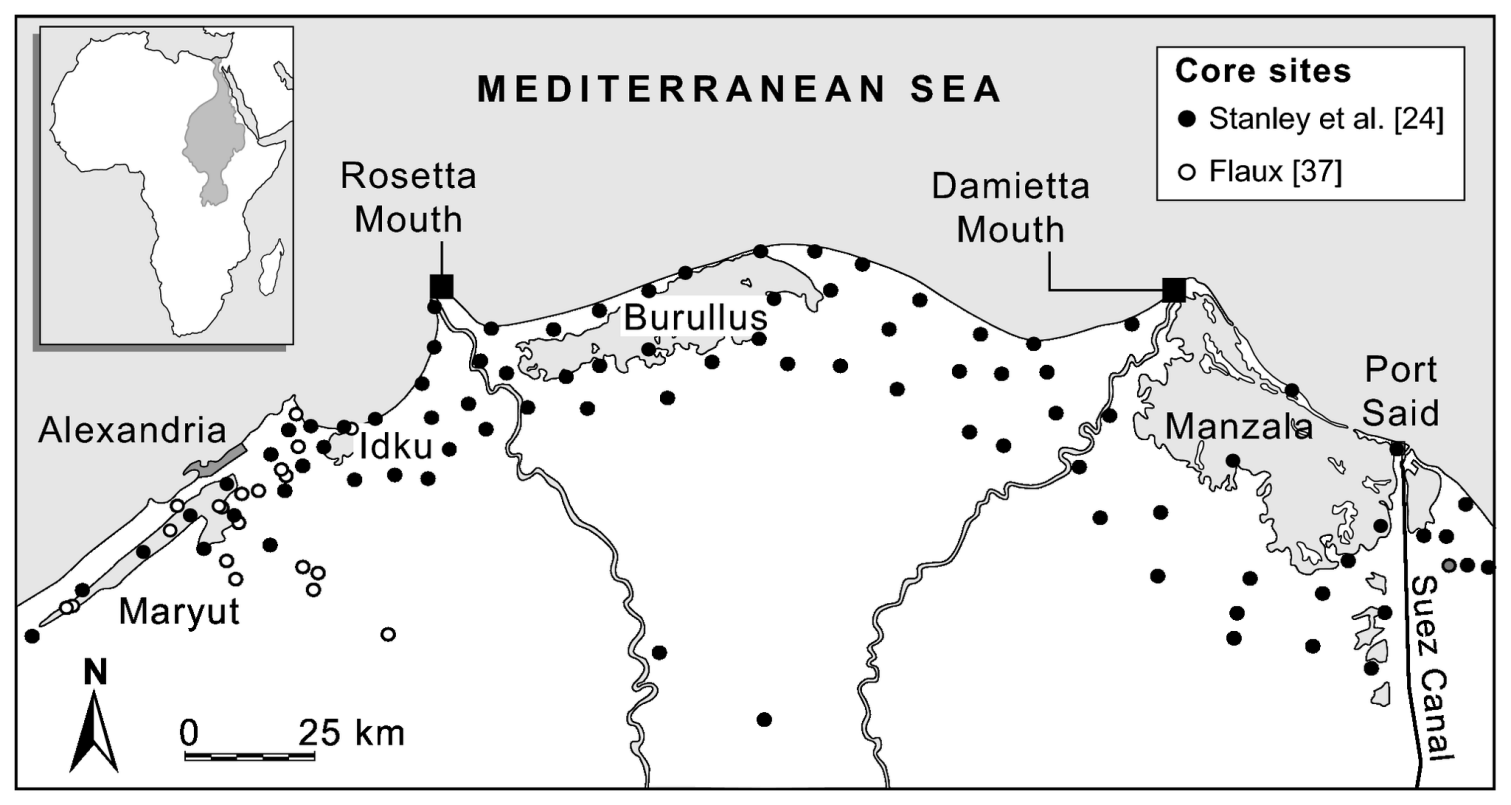
Location of the Nile Delta core sites used in this study.

To assess the Nile Delta’s state of health over centennial to millennial timescales, we generated a well-resolved 8000-year sediment accretion record using more >100 cores studded across the present deltaic fringe (Figure 2). This holistic reconstruction provides insights into deltaic response to climate change and human impacts from the early Holocene up to present day. At the regional scale, deltaic growth has been controlled by the combined interaction of sediment supply, sea-level rise and subsidence. These three histories have been used to generate the sediment accretion record.

## Materials and Methods

Various literature sources [23,24,34,35] and our present ongoing research [36-38] were used to compile a database of Holocene radiocarbon dates and stratigraphies from the Nile Delta area. A total of 359 radiocarbon entries were made in the database. Locations of core sites and sections (n = 105) are given in Figure 2. These have all been benchmarked relative to present Mean Sea Level (MSL). All radiocarbon determinations were standardized and calibrated using Oxcal [39] with the IntCal09 and Marine09 datasets [40].

Spatially averaged sedimentation rates were calculated for all radiocarbon couplets, using classic age-depth techniques. We refer the reader to [27] for further details. A matrix in annual increments was plotted for all sedimentation pairs, based on the age range of the radiocarbon calibrations. We subsequently summed annual increments and divided by the population present in each year to generate a spatially averaged sedimentation figure for the whole delta area. Data were smoothed using a smoothing spline. We stress that this is a holistic spatial average for the entire deltaic margin. We have not focused upon regional differences that might result, for instance, from fluvial avulsions or channel abandonment. The scientific data are supported by independent studies from the prodelta area [26] and Nile delta sedimentation rates before the construction of the Aswan High Dam. For example, the most recent figures (160 mm/century) of our reconstruction fit tightly with pre-1964 measurements of deltaic accretion [41].

**Figure 3:**
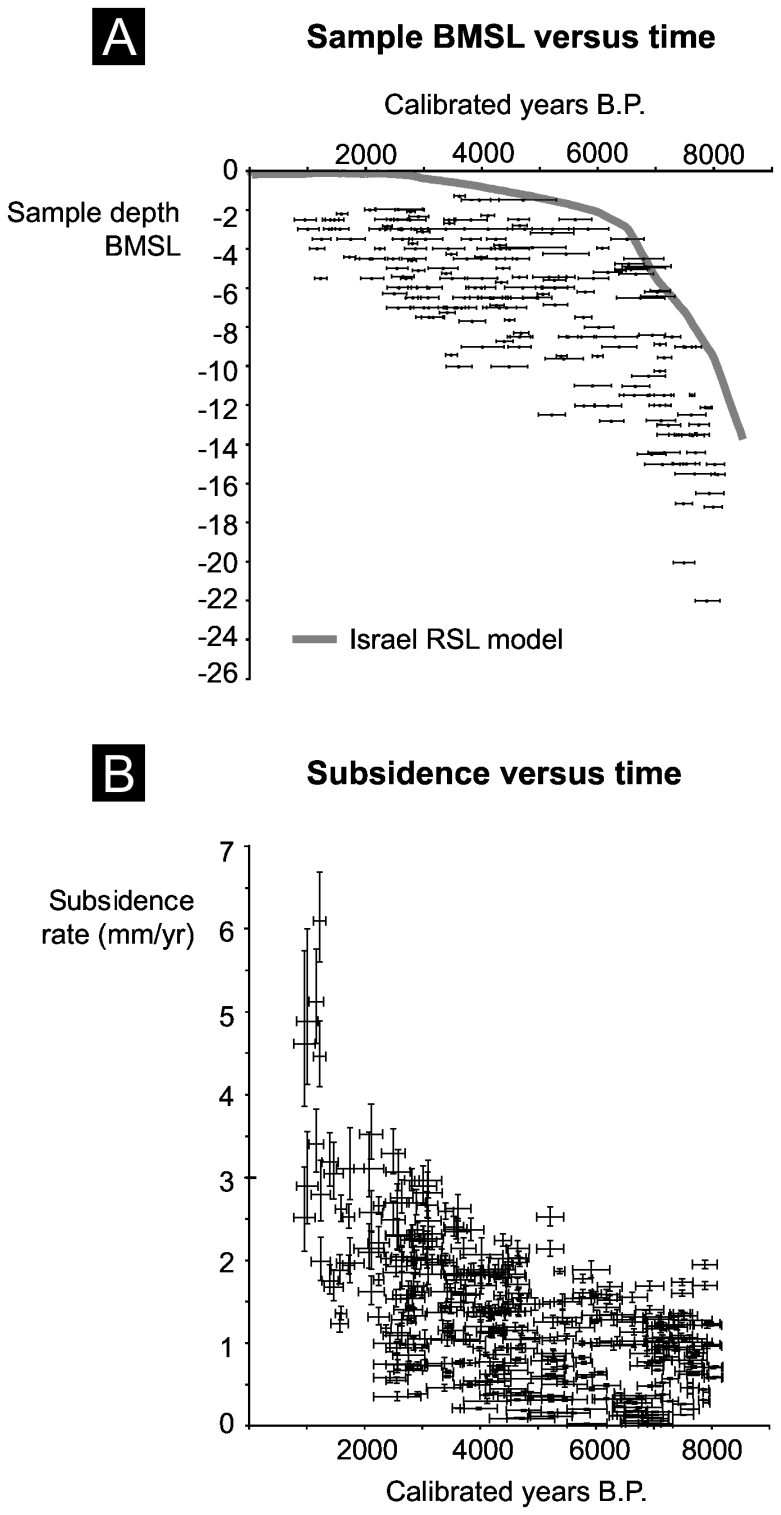
Data used to generate our Nile Delta subsidence history: (A) sample depth below MSL versus time; and (B) subsidence values. Because subsidence values are temporal (i.e. Holocene) and spatial averages, we stress that they are conservative (<2 mm/yr) when compared, for instance, with present rates (>5 mm/yr) around the Damietta lobe [31]. This suggests that surficial deltaic sediments undergo their most rapid phase of volume loss in the decades/centuries after deposition.

The subsidence history was generated using 194 radiocarbon dates deriving from organic-rich peat and lagoon deposits [23,24,36-38,42-44] (Figure 3). Prodelta muds and sublittoral sand deposits were not included in our analyses because these facies are subject to large altitudinal uncertainties. Peat and lagoon deposition is assumed to have occurred near historic mean sea level for each specimen. GPS and topographic maps were used to attitudinally benchmark these delta points relative to present Mean Sea Level (MSL). To investigate changes in Holocene delta elevation age-dependent predictions were obtained for the Relative Sea Level (RSL) of each point using model data from [45,46]. Elevation residuals were calculated as being the difference between the age of dated peat and lagoon deposits and concomitant modelled RSL. This generated 194 residual estimates for the magnitude of subsidence since deposition of the radiocarbon-dated point.

**Figure 4:**
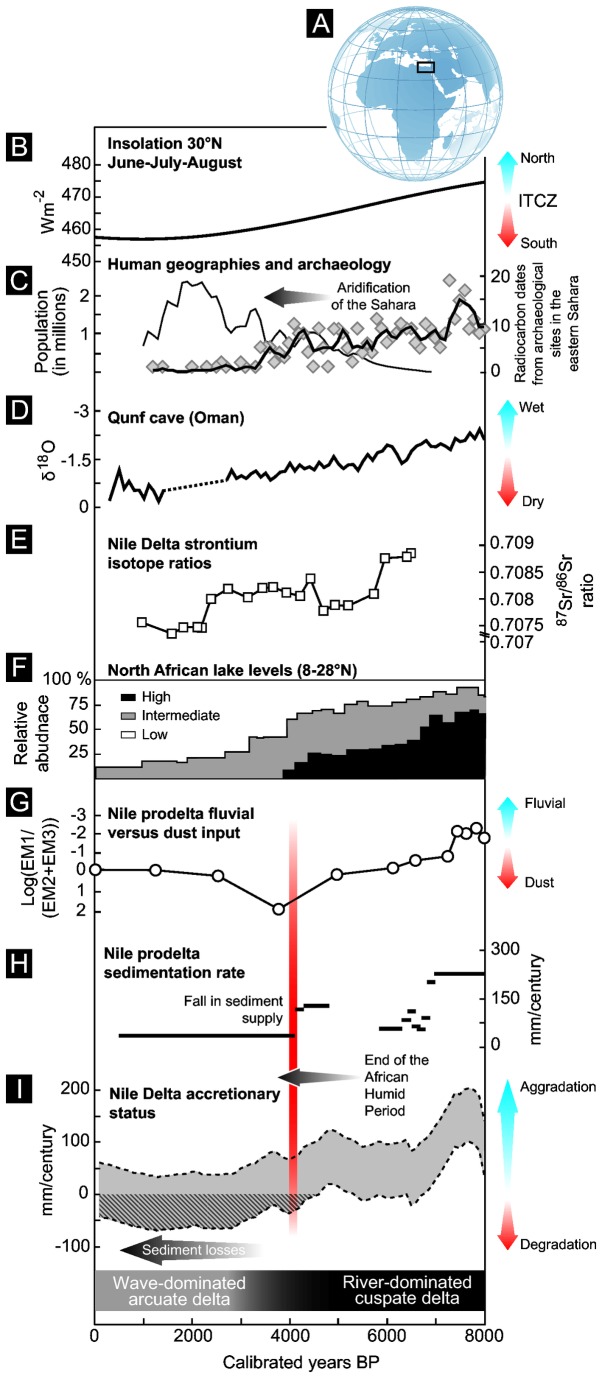
Nile Delta sediment accretion status and comparison with other regional proxies. (A) Location of the study area. (B) Holocene insolation at 30° N for June–July-August [68]. (C) On the right axis: radiocarbon dates from early and mid-Holocene occupation sites in the Eastern Sahara [48]. The solid black line denotes a three-point moving average. On the left axis: estimates for the population of the Nile valley in Egypt [29]. These are represented by the thin black line. (D) Holocene oxygen isotope record from the Arabian peninsula [25]. (E) Strontium isotope record from Manzala lagoon, eastern Nile Delta [69]. (F) North African lake levels [70,71]. (G) Reconstruction of changes in Nile runoff using the EM1/(EM2+EM3) ratio (record from [28]). (H) Nile prodelta sedimentation rates for the Holocene [26]. (I) Nile Delta accretionary status.

All three histories have been normalized into 100-year non-overlapping windows to generate the final time series in century^-1^. Estimates of the delta’s Holocene accretion status were calculated by subtracting spatially averaged sedimentation rates from Holocene averaged subsidence and modelled sea-level rise. Figure 4I plots the 20 and 80 percentiles of the subsidence history, which captures the most representative part of the dataset.

## Results and Discussion

The reconstructed sediment accretion history is presented in Figure 4I. For the past 8000 years, the values present a range of ~170 mm/century, with a gradual >70% decrease in accretionary status between ~7700 cal. BP and ~1300 cal. BP. This record is comparable in amplitude and direction to other regional palaeoclimate archives [25-27] and demonstrates that Nile Delta sedimentation has primarily been controlled by Holocene shifts in fluvial discharge, modulated by low-latitude summer insolation and the position of the eastern African Rain Belt [28]. During the early Holocene, increased summer insolation generated more intense monsoonal precipitation associated with high erosional activity which yielded significant sediment supply to the Nile depocenter that infilled the late Pleistocene topography [23]. This phase of high Nile flow and sediment delivery outpaced sea-level rise and subsidence, leading to the rapid growth of a fluvial-dominated cuspate delta, with progradation rates of up to 10 m/yr [47], characterized by the development of extensive wetland areas.

Around 5000 cal. BP, the accretion record presents decreases in sediment pulsing to the delta driven by a southerly migration of moisture-bearing monsoon rains (mean summer maximum ~15° N), which impacted upon both the watershed’s geosystems and the geography of human occupation along the Nile corridor and its bordering regions [48]. The end of the so-called African Humid Period has attracted considerable research interest due to its importance in assessing and identifying processes associated with transitional periods between climatic extremes [49]. Its origin, pattern and underlying forcing agents are not wholly understood, with apparent geographic disparities in the timing and amplitude of environmental changes across North Africa. Opposing scenarios of gradual insolation-driven aridification [50] versus one of rapid change in environmental conditions [51,52] have divided recent literature [53]. Whilst the linear response of the Nile Delta’s accretion status to insolation changes is unequivocal (r^2^ = 0.92), a series of centennial-scale fluctuations evoke shorter-scale, non-precessional forcing agents as pacemakers of deltaic change. These include, for instance, relative sea-level changes and ENSO variability [27]. Our reconstruction demonstrates that, for the Nile Delta, pronounced decreases in sediment supply began ~5000 years ago reaching a critical threshold ~4000 years ago. Beyond this tipping point, sediment losses imply that the delta became more vulnerable to erosion by extreme climate events, such as high-magnitude storms and floods, and sea-level rise. During the mid-Holocene, it has been proposed that the decrease in summer insolation and a concomitant increase in autumn insolation engendered a fall in the intensity of Blue Nile discharge and perhaps a relative increase in White Nile runoff [28]. Because the Blue Nile generates most of the sediment load to the Nile Delta (~70% at present) this shift manifestly had a significant impact upon the base-level depocentre.

Deltaic archives bear the signatures of climate-induced modifications in delta-front geomorphology around this time [36]. A strontium isotope record from the Maryut lagoon on the northwestern delta, for example, presents a striking increase in marine inputs after 5000 years ago [37] consistent with the erosion of protective beach ridges and salt water intrusion in a context of falling sediment supply. On the Nile Delta fan a pronounced increase in dust input, coupled with a decrease in the relative intensity of discharge from the Blue Nile, are consistent with the culmination of the gradual aridification of the Nile valley (Figure 4G [28]). The 4000 cal. BP decrease in sediment supply is also observed in other East African palaeoclimate archives [54,55], including the desiccation of Nile-fed Lake Faiyum [56], and historical evidence. An important inscription on the tomb of Ankhtifi, a nomarch during the early First Intermediate Period, narrates great famines that affected Egyptian populations around 4200-4050 cal. BP [57] and attests to the impact of hydroclimatic stress on agricultural production at this time. The failure of Nile floods suggests that the delta experienced pronounced change, possibly within the timeframe of just a few human generations. A rapid ~70% drop in Nile prodelta sedimentation rates confirms that this transition was relatively abrupt [26].

The onset of the mass balance losses coincides with an intensification of human occupation of the Nile Delta, when failing rains in the ‘Green’ Sahara focused populations along the Nile corridor [48,58,59]. This increased aridity probably enhanced fluvial incision and drainage of previously swampy areas in proximity to the main Nile channel, particularly conducive to the development of agriculture [60]. The delta area was attractive due to its high productivity and rich biodiversity, and rapidly became a focal point of social, cultural, and economic development for Egyptian civilization [29]. Archaeological excavations across the Nile Delta attest to a rich history of human occupation, with more than 700 sites having been identified to date [61,62]. Although chronological constraints are poor for many of these sites, it appears that between 6000 to 5000 years ago, Pre- and Proto-Dynastic occupation of the deltaic plain was preferentially centred on inherited topographic mounds that overlook the floodplain, locally known as ‘koms’ [37]. These topographic highs of Pleistocene age afforded protection from the Nile’s annual floods, the intensity of which is inferred to have been greater at this time [27]. At the onset of the Early Dynastic phase, around 5000 years ago, changes in the delta landscape, associated with a fall in Nile flow and seasonally receding flood zones, may have been one of the factors that encouraged the expansion of human activities onto the deltaic plain by altering ecological structure and resource availability [63]. The absence of robust chronological constraints precludes the precise spatio-temporal mapping of this process but nonetheless constitutes an interesting avenue for future research. This was a dynamic period of human cultural evolution on the Nile Delta and an intensification of human exploitation of the deltaic plain fostered channelling and drainage of the wetlands for irrigation agriculture, that modified sediment and water routing through the delta [64]. In a context of decreasing Nile flow, these ancient water-management technologies probably exacerbated the geosystem’s long-term fragility by promoting subsidence of Holocene deposits through groundwater lowering and microbial oxidation of organic-rich sediments. An intensification of human exploitation shows that, despite the ecosystems’ vulnerability to coastal change, the environmental potentiality of the Nile Delta (high fertility, resource multiplicity and transport capabilities) greatly outweighed perceived risks.

## Conclusions

This Nile Delta sediment accretion record reveals new insights into the delta’s long-term vulnerability to climate change and human impacts. We have documented a >70% decrease in the Nile Delta’s accretion status between the early and late Holocene, consistent with a gradual decrease in climate-driven sediment supply. The precise spatial expression of these sediment losses invites closer scrutiny (e.g. chronologies and geographies of avulsions, channel abandonment and pronounced areas of erosion). At the regional scale, the Nile Delta experienced a geomorphological restructuring from a river-dominated cuspate delta to a wave-dominated arcuate delta following the end of the ‘African Humid Period’. A corollary of the millennial-scale ebb in Nile flow and human channelling of the delta was a gradual reduction in the number of fluvial distributaries from up to nine around 5000 years ago to just two at present. Furthermore, we suggest that reduced river flow linked to a weakening of the Ethiopian Monsoon was particularly conducive to the expansion of human exploitation of the delta by exposing productive floodplain lands and producing a sharper contrast between seasonally-driven Nile minima and maxima. In terms of risks and vulnerabilities, the impact of sediment deficits would have been particularly acute along coastal margins and in areas of young Holocene strata, where subsidence rates in excess of 5 mm/yr are presently attested [31]. Although the current predicament of the Nile Delta - starved of significant sediment input since the completion of the Aswan High Dam - is without precedent for the Holocene, these new mass balance data suggest that the Nile Delta has a particularly long history of degradation by climate-driven changes in sediment delivery and human impacts.

## Supporting Information

File S1 2 figures and 1 table. Figure S1. Rank diagram of the 318 radiocarbon dates and calibrations included in the statistical analyses. The 2 sigma error bars of calibrations are denoted. Figure S2. Example of the calculation of sedimentation rates from a Maryut lagoon core. Table S1. Statistical correlation of Nile Delta accretion rates versus other archaeological and palaeoclimate proxies from the Nile valley and neighbouring regions.
(DOC)
