# Supplementary material for: Tracking Nile Delta Vulnerability to Holocene Change
Source: PLoS One. 2013 Jul 29;8(7):e69195. doi: 10.1371/journal.pone.0069195 (PMC3726729; doi:10.1371/journal.pone.0069195)
Supplement: File S1 — Figure S1. Rank diagram of the 318 radiocarbon dates and calibrations included in the statistical analyses. The 2 sigma error bars of calibrations are denoted. Figure S2. Example of the calculation of sedimentation rates from a Maryut lagoon core. Table S1. Statistical correlation of Nile Delta accretion rates versus other archaeological and palaeoclimate proxies from the Nile valley and neighbouring regions. (DOC) [file pone.0069195.s001.doc]

**Supporting Information**

**1. Nile database:** We compiled a database of all late Quaternary radiocarbon dates from the Nile Delta area using various literature sources and our present research [1-7] (see Figure 2). Locations of core sites and sections (n = 105) are given in Figure 2. To standardize the different data sources we structured results into a number of tabular fields based on stratigraphy, geographic coordinates, position relative to MSL and so forth. A total of 359 entries were made in the database; 41 of these dates were judged to have been reworked and subsequently excluded from further analyses. A rank diagram of the 318 radiocarbon dates and calibrations included in the statistical analyses is given in SI Figure 1. To standardize all radiocarbon dates, determinations were calibrated using Oxcal [8] with the IntCal09 and Marine09 datasets [9]. All calibrations are quoted at the two-sigma confidence range.


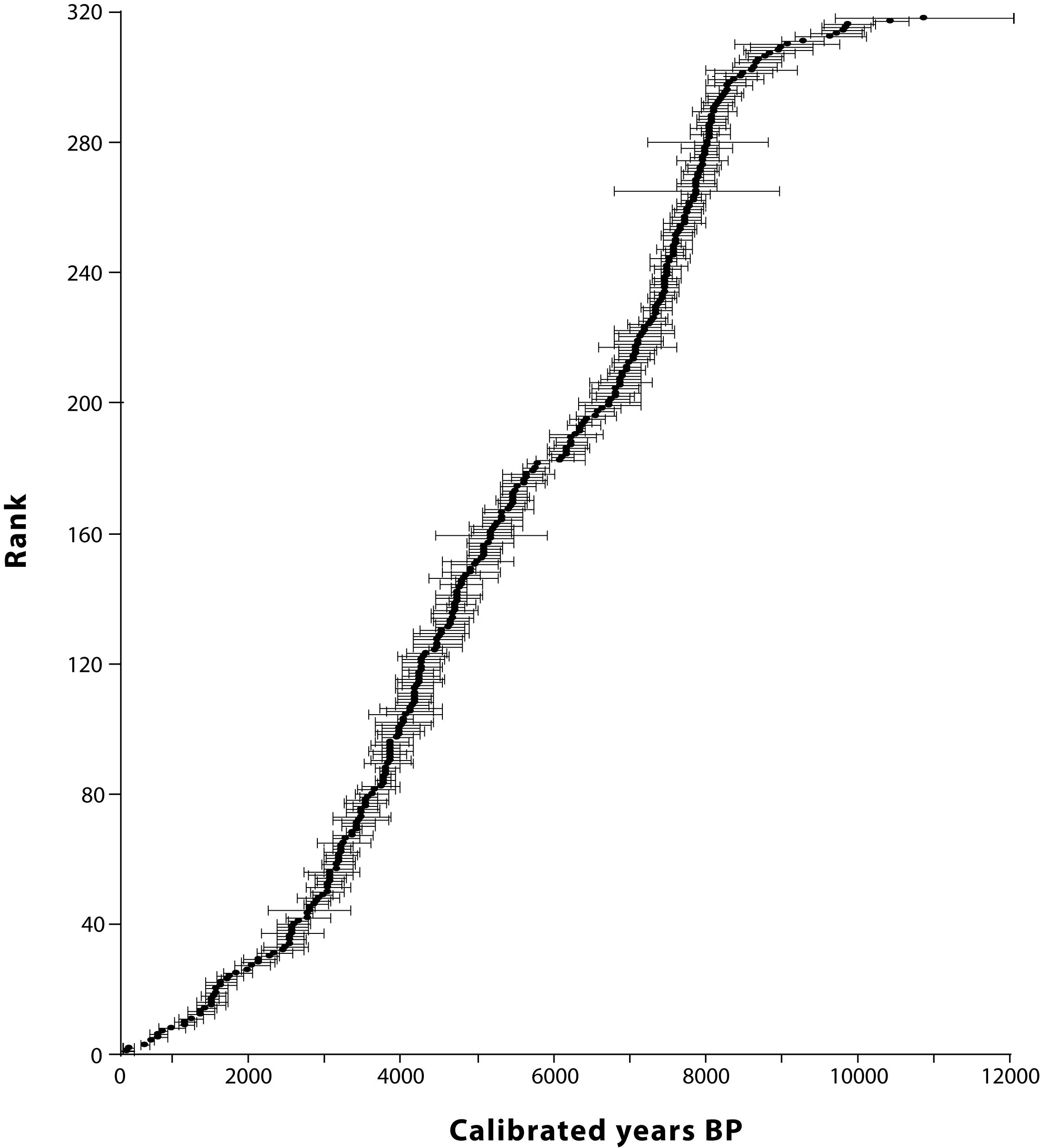


**SI Figure 1:** Rank diagram of the 318 radiocarbon dates and calibrations included in the statistical analyses. The 2 sigma error bars of calibrations are denoted.

**2. Nile sedimentation rates:** Spatially averaged sedimentation rates were calculated for all radiocarbon couplets using the classic age-depth technique (SI Figure 2). A matrix in annual increments was plotted for all sedimentation pairs in the dataset. We subsequently summed annual increments and divided by the population present in each year to generate a spatially averaged sedimentation figure for the whole delta area. All rates were summed in 100-year non-overlapping windows to generate the final time series in century^-1^.


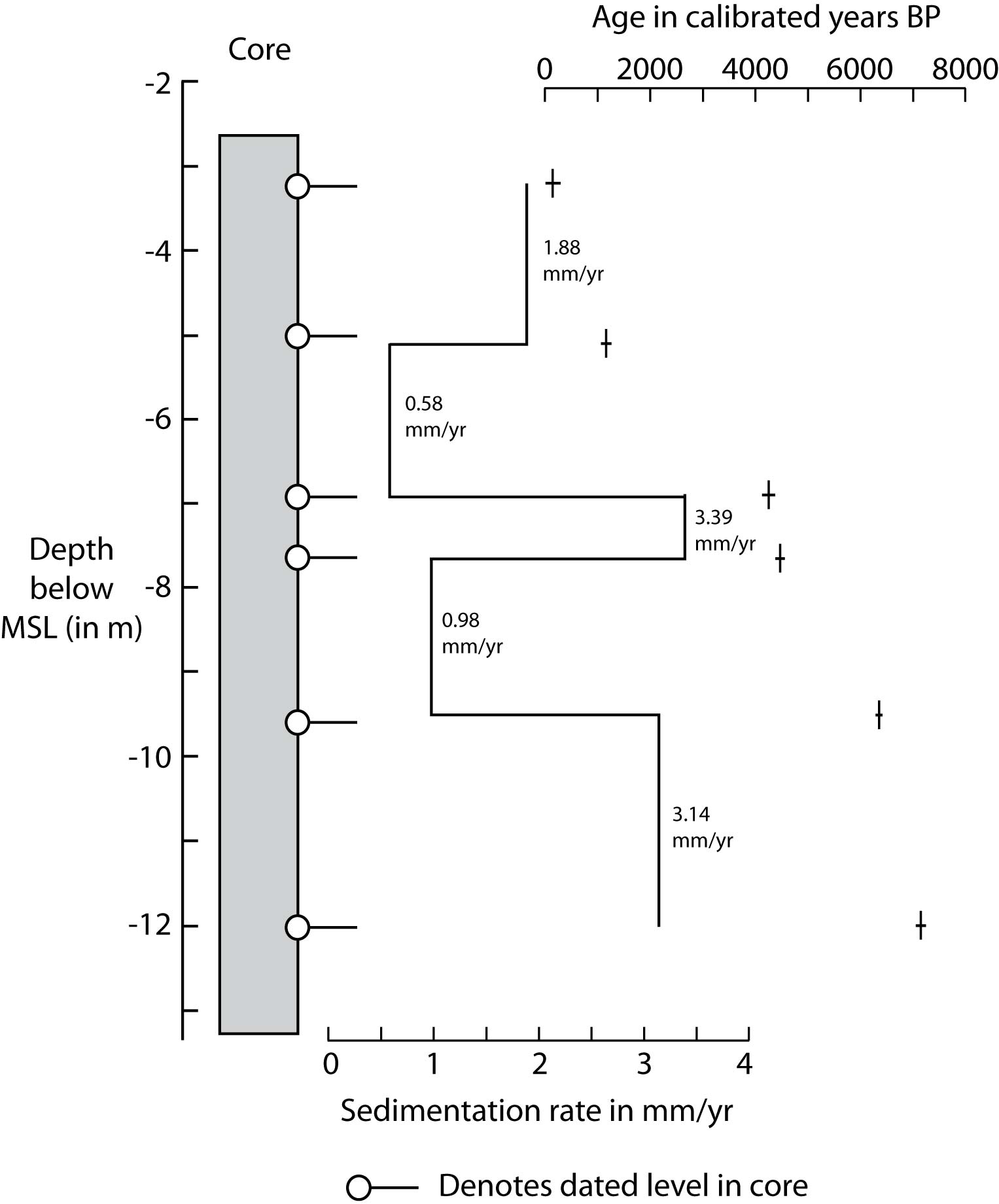


**SI Figure 2:** Example of the calculation of sedimentation rates from a Maryut lagoon core [see 7].

**3. Subsidence and sea-level histories:** we isolated a subset of 194 radiocarbon dates deriving from organic-rich peat and lagoon deposits (Figure 3A). To probe changes in Holocene delta elevation we obtained age-dependent predictions for the RSL of each point using model data from [10] and [11]. It is assumed that eustatic and glacio-hydro-isostatic signals have been uniform in time and space across the delta. 194 residual estimates for the magnitude of subsidence since deposition of the radiocarbon-dated point were calculated by subtracting the age of dated peat and lagoon deposits from concomitant modeled RSL. The 20 (57 mm/century) and 80 (193 mm/century) percentiles of this subsidence history were used to generate Figure 4I. These subsidence rates are consistent with Holocene rates from other mega-deltas.

Estimates of the delta’s Holocene accretionary status were calculated by subtracting spatially averaged sedimentation rates from the sum of Holocene averaged subsidence and modelled sea-level rise

**4. Comparison with palaeoclimatological archives and human occupation**

We compared and contrasted our data with a number of other palaeoclimatological archives. Records were normalized by averaging data into 100-year non-overlapping windows.

(a) *Insolation*. Insolation quantities (W/m^2^) in 100-year windows derived from the orbital and precessional quantities [12] were downloaded at:

<http://www.imcce.fr/Equipes/ASD/insola/earth/online/index.php>. Simulations are reported for 30°N.

(b) *Egypt and Sudan Saharan archaeological data*. Archaeological radiocarbon data from the deserts of Libya, Egypt, Sudan and Chad were obtained from [13]. We recalibrated data in Oxcal and used the µ function to sort dates into non-overlapping 100-year windows. Nile valley population estimates in Egypt derive from [14].

(c) *Qunf Cave, Oman* stalagmite record [15,16]. Raw data for plotting and statistical analyses were downloaded from the NOAA website at: <http://www.ncdc.noaa.gov/paleo/metadata/noaa-cave-5541.html>. δ^18^O data were compared and contrasted with Nile sedimentation rates. Because of the inverse relationship between δ^18^O and the amount of precipitation in regions affected by monsoons, δ^18^O scales have been plotted high to low for comparative purposes with the Nile record.

(d) The Nile Delta the strontium isotope curve derives from [17].

(e) The Nile flow proxies derive from a Nile fan record (Marine sediment core P362/2-33; 31°40.51N; 29°45.00E)) published by [18]. Statistically meaningful end-members (EMs) were calculated using the particle-size measurements and the end-member model algorithm (EMMA). See [18] for further details.

(f) Nile prodelta sedimentation rates for the Holocene are from [19].

**5. Statistical analyses**

To test the statistical strength of our Nile accretion record with other regional proxies we used Cross-Correlation Analysis. Cross-Correlation Analysis (CCA; P=0.05) is a generalization of detrended fluctuation analysis and is based on detrended covariance. To quantify the power-law cross-correlations in nonstationary time series (see proxy series above), we have considered two long-range cross-correlated time series of equal length. The cross correlation is plotted as a function of alignment position. Positive correlation coefficients are considered, focussing on the Lag 0 value (with ca. +.50 as significant threshold). Negative correlations are also assessed to test the inverse- or non-correlation between the two time-series (with ca. -.50 as significant threshold). Null values indicate the absence of a correlation. Six of the seven time series are above the significant correlation threshold of + or - .7.

|  | CCA |
| --- | --- |
| Insolation 30°N JJA average [12] | 0.95728 |
| Archaeology Sahara [13] | 0.82915 |
| Nile population Egypt [14] | -0.85704 |
| Qunf cave [15] | -0.90602 |
| Nile Delta strontium isotope record [17] | 0.718 |
| Nile prodelta fluvial versus dust input [18] | -0.66543 |
| Nile prodelta sedimentation [19] | 0.87462 |

**SI Table 1:** Statistical correlation of Nile Delta accretion rates versus other archaeological and palaeoclimate proxies from the Nile valley and neighbouring regions.

**SI references**

1. Sneh A, Weissbrod T, Ehrlich A, Horowitz A, Moshkovitz S et al. (1986) Holocene evolution of the northeastern corner of the Nile Delta. Quaternary Research 26: 194-206.

2. Wunderlich J (1989) Untersuchungen zur Entwicklung des westlichen Nildeltas im Holozän. Marburger Geographische schriften.

3. Stanley J-D, Warne AG (1993) Nile Delta: Recent geological evolution and human impact. Science 260: 628–634.

4. Stanley J-D, McRea JE, Waldron JC (1996) Nile Delta Drill Core and Sample Database for 1985–1994: Mediterranean Basin (MEDIBA) Program. Washington, DC: Smithsonian Contributions to the Marine Sciences, No. 37, Smithsonian Institution Press.

5. Stanley J-D, Toscano MA (2009) Ancient Archaeological Sites Buried and Submerged along Egypt’s Nile Delta Coast: Gauges of Holocene Delta Margin Subsidence. Journal of Coastal Research 25: 158-170.

6. Flaux C, Morhange C, Marriner N, Rouchy J-M (2011) Balance entre les influences marines et nilotiques dans le budget hydrologique de la lagune du Mariout (delta du Nil) entre 7800 et 3000 cal. BP. Géomorphologie 3: 261-278.

7. Flaux C (2012) Holocene palaeo-environments of the Maryut lagoon in the NW Nile Delta, Egypt [PhD thesis]. Aix-en-Provence: Aix-Marseille Université.

8. Bronk Ramsey C (2000) OxCal Program v3.5: The Manual.

9. Reimer PJ, Baillie MGL, Bard E, Bayliss A, Beck JW et al. (2009) IntCal09 and Marine09 radiocarbon age calibration curves, 0-50,000 years cal. BP. Radiocarbon 51: 1111-1150.

10. Sivan D, Wdowinski S, Lambeck K, Galili E, Raban A (2001) Holocene sea-level changes along the Mediterranean coast of Israel, based on archaeological observations and numerical model. Palaeogeography, Palaeoclimatology, Palaeoecology 167: 101-117.

11. Sivan D, Lambeck K, Toueg R, Raban A, Porath Y et al. (2004) Ancient coastal wells of Caesarea Maritima, Israel, an indicator for relative sea level changes during the last 2000 years. Earth and Planetary Science Letters 222: 315-330.

12. Laskar J, Robutel P, Joutel F, Gastineau M, Correia ACM et al. (2004) A long term numerical solution for the insolation quantities of the Earth. Astronomy & Astrophysics 428: 261-285.

13. Kuper R, Kröpelin S (2006) Climate-controlled Holocene occupation of the Sahara: motor of Africa’s evolution. Science 313: 803-807.

14. Butzer KW (1976) Early Hydraulic Civilization in Egypt: a Study in Cultural Ecology. Chicago: University of Chicago Press.

15. Fleitmann D, Burns SJ, Mudelsee M, Neff UN, Kramers J et al. (2003) Holocene forcing of the Indian monsoon recorded in a stalagmite from Southern Oman. Science 300: 1737–1739.

16. Fleitmann D, Burns SJ, Mangini A, Mudelsee M, Kramers J et al. (2007) Holocene ITCZ and Indian monsoon dynamics recorded in stalagmites from Oman and Yemen (Socotra). Quaternary Science Reviews 26: 170–188.

17. Krom MD, Stanley JD, Cliff RA, Woodward JC (2002) Nile river fluctuations over the past 7000 yrs and their key role in sapropel development. Geology 30: 71–74.

18. Blanchet CL, Tjallingii R, Frank M, Lorenzen J, Reitz A et al. (2013) High- and low-latitude forcing of the Nile River regime during the Holocene inferred from laminated sediments of the Nile deep-sea fan. Earth and Planetary Science Letters 364: 98-110.

19. Revel M, Ducassou E, Grousset FE, Bernasconi SM, Migeon S et al. (2010) 100,000 Years of African monsoon variability recorded in sediments of the Nile next term margin. Quaternary Science Reviews 29: 1342–1362.
